# Effects of Acute Hypernatremia on the Electrophysiology of Single Human Ventricular Cardiomyocytes: An *In Silico* Study

**DOI:** 10.31083/j.rcm2506194

**Published:** 2024-05-28

**Authors:** Arie O. Verkerk, Ronald Wilders

**Affiliations:** ^1^Department of Medical Biology, Amsterdam Cardiovascular Sciences, Amsterdam UMC, University of Amsterdam, 1105 AZ Amsterdam, The Netherlands; ^2^Department of Experimental Cardiology, Heart Center, Amsterdam Cardiovascular Sciences, Amsterdam UMC, University of Amsterdam, 1105 AZ Amsterdam, The Netherlands

**Keywords:** hypernatremia, sodium, cardiomyocytes, action potential, ion currents, human, heart, ventricle, computer simulations, hyperosmosis

## Abstract

**Background:**

Clinical and experimental data on the cardiac effects of acute hypernatremia are scarce and inconsistent. We aimed to determine and understand the effects of different levels of acute hypernatremia on the human ventricular action potential.

**Methods:**

We performed computer simulations using two different, very comprehensive models of the electrical activity of a single human ventricular cardiomyocyte, *i.e.*, the Tomek–Rodriguez model following the O’Hara–Rudy dynamic (ORd) model and the Bartolucci–Passini–Severi model as published in 2020 (known as the ToR-ORd and BPS2020 models, respectively). Mild to extreme levels of hypernatremia were introduced into each model based on experimental data on the effects of hypernatremia on cell volume and individual ion currents.

**Results:**

In both models, we observed an increase in the intracellular sodium and potassium concentrations, an increase in the peak amplitude of the intracellular calcium concentration, a hyperpolarization of the resting membrane potential, a prolongation of the action potential, an increase in the maximum upstroke velocity, and an increase in the threshold stimulus current at all levels of hypernatremia and all stimulus rates tested. The magnitude of all of these effects was relatively small in the case of mild to severe hypernatremia but substantial in the case of extreme hypernatremia. The effects on the action potential were related to an increase in the sodium–potassium pump current, an increase in the sodium–calcium exchange current, a decrease in the rapid and slow delayed rectifier potassium currents, and an increase in the fast and late sodium currents.

**Conclusions:**

The effects of mild to severe hypernatremia on the electrical activity of human ventricular cardiomyocytes are relatively small. In the case of extreme hypernatremia, the effects are more pronounced, especially regarding the increase in threshold stimulus current.

## 1. Introduction

Under normal conditions, serum ${\mathrm{Na}}^{+}$ concentrations in the human body are finely maintained within a narrow range of 135–145 mmol/L despite large fluctuations in water or salt intake [1]. Nevertheless, hyponatremia and hypernatremia are relatively common electrolyte disorders [1, 2]. Minor abnormalities in ${\mathrm{Na}}^{+}$ levels are common and rarely of clinical significance; however, more severe ${\mathrm{Na}}^{+}$ disorders are still relatively common and are very frequently clinically significant [3, 4, 5]. Hypernatremia, which is much less common than hyponatremia, is generally defined as serum ${\mathrm{Na}}^{+}$ levels >145 mmol/L [1]. The degree of hypernatremia is generally considered mild when serum ${\mathrm{Na}}^{+}$ levels are between 145 and 155 mmol/L and severe when serum ${\mathrm{Na}}^{+}$ levels are >155 mmol/L [2]. Somewhat surprisingly, the exact boundaries of what is considered the normal range of serum ${\mathrm{Na}}^{+}$ levels vary from institution to institution, as do the boundaries between mild and severe hypernatremia [2, 6, 7]. When serum ${\mathrm{Na}}^{+}$ levels exceed 190 mmol/L, the hypernatremia becomes extreme [8, 9]. Case reports have reported ${\mathrm{Na}}^{+}$ levels ranging from 207 to 255 mmol/L [10, 11, 12, 13, 14, 15].

It is well known that hypernatremia, especially when acute and severe, can induce brain shrinkage that may cause vascular rupture and cerebral bleeding, resulting in permanent neurologic damage or even death [10, 16, 17, 18]. Much less is known about the effects of acute hypernatremia on the heart and the underlying mechanisms. Data on the direct effects of acute hypernatremia on individual membrane currents of cardiac myocytes are lacking. However, some data exist on the changes in individual membrane currents of cardiac myocytes that are acutely exposed to hyperosmotic solutions (induced by the addition of sucrose or mannitol to the extracellular solution rather than hypernatremia). Such exposure is associated with a rapid shrinkage of the cells through the loss of intracellular water. This shrinkage occurs within 2–3 minutes and is fully reversible [19, 20, 21, 22]. By exposing isolated guinea pig ventricular cardiomyocytes to hyperosmotic Tyrode’s solution with a 1.5 times normal osmolarity, Ogura *et al*. [21] observed decreases in cell volume of 19 ± 2% (mean ± standard error of the mean, SEM, *n* = 8) and of ≈20% [22]. A reduction of 19 ± 2% (mean ± SEM, *n* = 8) was also observed by Missan *et al*. [23], who also exposed isolated guinea pig ventricular cardiomyocytes to hyperosmotic Tyrode’s solution with a 1.5 times normal osmolarity. In isolated rat ventricular cardiomyocytes, ≈18% of the cell volume is osmotically inactive [24]. In the rabbit, this is ≈34% [19], and in the guinea pig it is ≈35% [21]. The decrease in cell volume, without affecting membrane capacitance [22], may, by itself, affect membrane currents by increasing the intracellular ion concentrations. However, cell shrinkage may also have direct functional effects on the proteins embedded in the cell membrane, including those of ion channels, pumps, and exchangers [22, 25]. The available data on such direct functional effects of acute exposure to a hyperosmotic solution on individual ion currents, as obtained in isolated cardiomyocytes or an expression system, are summarized in Table 1 (Ref. [20, 22, 23, 25, 26, 27, 28, 29]).

**Table 1.** **Effects of an acute exposure to a hyperosmotic solution on individual cardiac ion currents**.

Current	Cell preparation	${\mathrm{Hyperosmolarity}}^{1}$	Observation	Study
$I_{\text{CaL}}$	Guinea pig ventricular cardiomyocytes	30%	No consistent change of $I_{\text{CaL}}$	Sasaki *et al*. [26]
	Guinea pig ventricular cardiomyocytes	50%	≈28% decrease in amplitude; slightly slowed inactivation	Ogura *et al*. [20]
	Rat ventricular cardiomyocytes	30%	≈27% increase in amplitude; slightly accelerated inactivation	Luo *et al*. [27]
$I_{\text{Kr}}$	Guinea pig ventricular cardiomyocytes	30%	≈44% decrease in amplitude	Sasaki *et al*. [26]
	Guinea pig ventricular cardiomyocytes	50%	≈30% decrease in amplitude	Ogura *et al*. [22]
	Chinese hamster ovary (CHO) cells stably expressing $I_{\text{Kr}}$ channels	40%	≈57% decrease in amplitude; no major effects on voltage dependence	Yabuuchi *et al*. [28]
$I_{\text{Ks}}$	Guinea pig ventricular cardiomyocytes	50%	≈50% decrease in amplitude	Ogura *et al*. [22]
	Guinea pig ventricular cardiomyocytes	50%	“Marked inhibition”	Missan *et al*. [23]
$I_{\text{K1}}$	Guinea pig ventricular cardiomyocytes	50%	No effect on $I_{\text{K1}}$	Missan *et al*. [23]
$I_{\text{NaCa}}$	Guinea pig ventricular cardiomyocytes	30%	≈23% increase in amplitude	Wright *et al*. [29]
$I_{\text{NaK}}$	Guinea pig ventricular cardiomyocytes	50%	≈40% decrease in amplitude	Whalley *et al*. [25]
	Guinea pig ventricular cardiomyocytes	30%	≈70% decrease in amplitude, although the decrease was only apparent in 15 of 29 experiments	Sasaki *et al*. [26]

$I_{\text{CaL}}$, L-type ${\mathrm{Ca}}^{\text{2+}}$ current; $I_{\text{Kr}}$, rapid delayed rectifier $K^{+}$ current; $I_{\text{Ks}}$, slow delayed rectifier $K^{+}$ current; $I_{\text{K1}}$, inward rectifier $K^{+}$ current; $I_{\text{NaCa}}$, ${\mathrm{Na}}^{+}$–${\mathrm{Ca}}^{\text{2+}}$ exchange current; $I_{\text{NaK}}$, ${\mathrm{Na}}^{+}$–$K^{+}$ pump current. ^1^Hyperosmosis induced by the addition of sucrose or mannitol to the extracellular solution.

The experimental data in Table 1 are required to construct a model for use in computer simulations of the effects of acute hypernatremia (and associated hyperosmosis) on the electrophysiology of single human ventricular cardiomyocytes, as detailed in the Materials and Methods section below. In the case of the rapid delayed rectifier $K^{+}$ current ($I_{\text{Kr}}$), the slow delayed rectifier $K^{+}$ current ($I_{\text{Ks}}$), and the ${\mathrm{Na}}^{+}$–$K^{+}$ pump current ($I_{\text{NaK}}$), the data from the different studies are largely consistent (Table 1). However, the experimental data for the L-type calcium ${\mathrm{Ca}}^{\text{2+}}$ current ($I_{\text{CaL}}$) from three studies seem inconsistent, at least at first glance. In the study by Ogura *et al*. [20], in which the intracellular ${\mathrm{Ca}}^{\text{2+}}$ concentration ([${\mathrm{Ca}}^{\text{2+}}$]_i_) was only moderately buffered, accompanying experiments with indo-1-loaded cardiomyocytes suggested that the decrease in $I_{\text{CaL}}$ amplitude was due to a rapid increase in [${\mathrm{Ca}}^{\text{2+}}$]_i_ rather than a direct functional effect on the $I_{\text{CaL}}$ channels. This is in line with the observations of Sasaki *et al*. [26], who observed “no consistent change in $I_{\text{CaL}}$” when using a pipette solution containing 5.0 mmol/L ethylene glycol-bis (β-aminoethyl ether)-N,N,N’,N’-tetraacetic acid (EGTA) or 10 mmol/L 1,2-bis (2-aminophenoxy)ethane-N,N,N’,N’-tetraacetic acid (BAPTA), thereby strongly buffering [${\mathrm{Ca}}^{\text{2+}}$]_i_. Thus, the findings of Ogura *et al*. [20] and Sasaki *et al*. [26] both suggest that there is no direct effect from the hyperosmosis (osmolarity 1.5 times normal) on the amplitude of $I_{\text{CaL}}$ and that the apparent discrepancy in their observations is largely due to the differences in the extent to which [${\mathrm{Ca}}^{\text{2+}}$]_i_ was buffered. However, Luo *et al*. [27], who used a pipette solution containing 10 mmol/L EGTA, thus also strongly buffering [${\mathrm{Ca}}^{\text{2+}}$]_i_, still showed an ≈27% increase in $I_{\text{CaL}}$ amplitude (osmolarity 1.3 times normal).

In 2011, O’Hara *et al*. [30] published a comprehensive model of the electrical activity of a single human ventricular cardiomyocyte, which has often been considered the “gold standard” for such a model over the past decade [31]. This O’Hara–Rudy dynamic (ORd) cell model has since been widely used in computer simulations of the electrical activity of a single human ventricular cardiomyocyte in health and disease. However, starting from the ORd model, both Tomek *et al*. [32] and Bartolucci *et al*. [31] developed novel, very comprehensive models of the electrical activity of a single human ventricular cardiomyocyte. These well-documented models were published in 2019 and 2020 and are known as the Tomek–Rodriguez model, following the ORd model (ToR–ORd model) and the Bartolucci–Passini–Severi model as published in 2020 (BPS2020 model), respectively. Although both models can be considered major updates of the ORd model, with many highly important improvements, they were developed along different lines, which makes it useful to run simulations with both models when, as we did in the present study, performing an *in silico* study of the effects of acute hypernatremia on the electrophysiology of single human ventricular cardiomyocytes.

## 2. Materials and Methods

The electrical activity of a single human ventricular cardiomyocyte was simulated using the comprehensive models of such a cell developed by Bartolucci *et al*. [31] and Tomek *et al*. [32]. These are known as the BPS2020 and ToR–ORd models, respectively. For the BPS2020 model, we used the CellML [33] code that the developers of the model made publicly available on the website of the MCBeng community of researchers in the field of Molecular and Cellular Bioengineering (https://www.mcbeng.it/en/; accessed on November 6, 2023). For the ToR–ORd model, we used the CellML code made publicly available by the model developers on the GitHub platform (https://github.com/jtmff/torord; accessed on November 7, 2023). The CellML code of the models was edited and run in version 0.9.31.1409 of the Windows-based Cellular Open Resource (COR) environment [34]. All simulations were run for a simulated period of 10 min, which appeared long enough to achieve steady-state or quasi-steady-state behavior at each level of hypernatremia. The data analyzed are from the final five seconds of this 10-minute period. Action potentials (APs) were elicited with a 1 ms, ≈2× threshold stimulus.

The experimentally observed changes in individual membrane currents in response to the 10–50% hyperosmotic extracellular solutions were incorporated as the scaling factors for $I_{\text{CaL}}$, $I_{\text{Kr}}$, $I_{\text{Ks}}$, the inward rectifier $K^{+}$ current ($I_{\text{K1}}$), $I_{\text{NaK}}$, and the ${\mathrm{Na}}^{+}$–${\mathrm{Ca}}^{\text{2+}}$ exchange current ($I_{\text{NaCa}}$) listed in Table 2. The discrepancy between the experimental data on $I_{\text{CaL}}$ by Ogura *et al*. [20] and Sasaki *et al*. [26] initially and then by Luo *et al*. [27] (see Introduction) was ignored by using a scaling factor of 1 for $I_{\text{CaL}}$ (*i.e.*, no hyperosmosis-induced—in the model case hypernatremia-induced—change in $I_{\text{CaL}}$). Other parameters of the cell models that are relevant in the setting of hypernatremia (and therefore listed in Table 2) are the extracellular concentrations of ${\mathrm{Ca}}^{\text{2+}}$, $K^{+}$, ${\mathrm{Na}}^{+}$, and ${\mathrm{Cl}}^{-}$, which are slightly different between the two models, and the cell volume.

**Table 2.** **Parameter settings in the BPS2020 and ToR–ORd human ventricular cell models**.

		Baseline	10% hypernatremia	20% hypernatremia	50% hypernatremia
Scaling factors					
	$I_{\text{CaL}}$ ^1^	1	1	1	1
	$I_{\text{Kr}}$ ^1^	1	0.920	0.840	0.600
	$I_{\text{Ks}}$ ^1^	1	0.900	0.800	0.500
	$I_{\text{K1}}$ ^1^	1	1	1	1
	$I_{\text{NaCa}}$ ^1^	1	1.077	1.153	1.383
	$I_{\text{NaK}}$ ^1^	1	0.867	0.733	0.333
BPS2020 model					
	[${\mathrm{Ca}}^{\text{2+}}$]_e_ (mmol/L)	2.7	2.7	2.7	2.7
	[$K^{\text{+}}$]_e_ (mmol/L)	5.4	5.4	5.4	5.4
	[${\mathrm{Na}}^{\text{+}}$]_e_ (mmol/L)	144	158.4	172.8	216
	[${\mathrm{Cl}}^{\text{−}}$]_e_ ${\mathrm{(mmol/L)}}^{2}$	154.8	169.2	183.6	226.8
	$V_{\text{cell}}$ ${\mathrm{(\%)}}^{3}$	100	94.17	89.25	78.28
ToR–ORd model					
	[${\mathrm{Ca}}^{\text{2+}}$]_e_ (mmol/L)	1.8	1.8	1.8	1.8
	[$K^{\text{+}}$]_e_ (mmol/L)	5.0	5.0	5.0	5.0
	[${\mathrm{Na}}^{\text{+}}$]_e_ (mmol/L)	140	154	168	210
	[${\mathrm{Cl}}^{\text{−}}$]_e_ (mmol/L)	150	164	178	220
	$V_{\text{cell}}$ ${\mathrm{(\%)}}^{3}$	100	94.14	89.21	78.21

$I_{\text{CaL}}$, L-type ${\mathrm{Ca}}^{\text{2+}}$ current; $I_{\text{Kr}}$, rapid delayed rectifier $K^{+}$ current; $I_{\text{Ks}}$, slow delayed rectifier $K^{+}$ current; $I_{\text{K1}}$, inward rectifier $K^{+}$ current; $I_{\text{NaCa}}$, ${\mathrm{Na}}^{+}$–${\mathrm{Ca}}^{\text{2+}}$ exchange current; $I_{\text{NaK}}$, ${\mathrm{Na}}^{+}$–$K^{+}$ pump current; [${\mathrm{Ca}}^{\text{2+}}$]_e_, extracellular ${\mathrm{Ca}}^{\text{2+}}$ concentration; [$K^{+}$]_e_, extracellular $K^{+}$ concentration; [${\mathrm{Na}}^{+}$]_e_, extracellular ${\mathrm{Na}}^{+}$ concentration; [${\mathrm{Cl}}^{-}$]_e_, extracellular ${\mathrm{Cl}}^{-}$ concentration; $V_{\text{cell}}$, cell volume; BPS2020, Bartolucci-Passini-Severi model as published in 2020; ToR–ORd, Tomek–Rodriguez model following the O’Hara–Rudy dynamic model. ^1^Scaling factors of 0.6 for $I_{\text{Kr}}$ and 0.5 for $I_{\text{Ks}}$ at 50% hypernatremia estimated from the experimental data listed in Table 1; scaling factors of 1.23 for $I_{\text{NaCa}}$ and 0.6 for $I_{\text{NaK}}$ at 30% hypernatremia estimated from the experimental data listed in Table 1; scaling factors for other degrees of hypernatremia obtained by linear interpolation or extrapolation. ^2^[${\mathrm{Cl}}^{-}$]_e_ not included in the BPS2020 model, but computed from the extracellular charge of ${\mathrm{Ca}}^{\text{2+}}$, $K^{+}$, and ${\mathrm{Na}}^{+}$ ions. ^3^$V_{\text{cell}}$ computed from the increase in extracellular osmolarity, assuming an osmotically inactive fraction of the cell volume of 32% [19, 21, 24].

## 3. Results

First, we carried out computer simulations that examined the effects of different levels of hypernatremia on the electrical activity of the BPS2020 model of a single human ventricular cardiomyocyte. APs were elicited at a rate of 50, 75, and 100 per minute, resulting in beating rates of 50, 75, and 100 beats per minute (bpm), respectively. Hypernatremia was simulated by increasing the extracellular concentration of sodium chloride by 10, 20, and 50%, corresponding to mild, severe, and extreme hypernatremia, respectively. Next, these simulations were repeated with the ToR–ORd model to assess to which extent the observed effects were model-dependent.

### 3.1 Effects of Hypernatremia in the BPS2020 Model

**Fig. 1.**
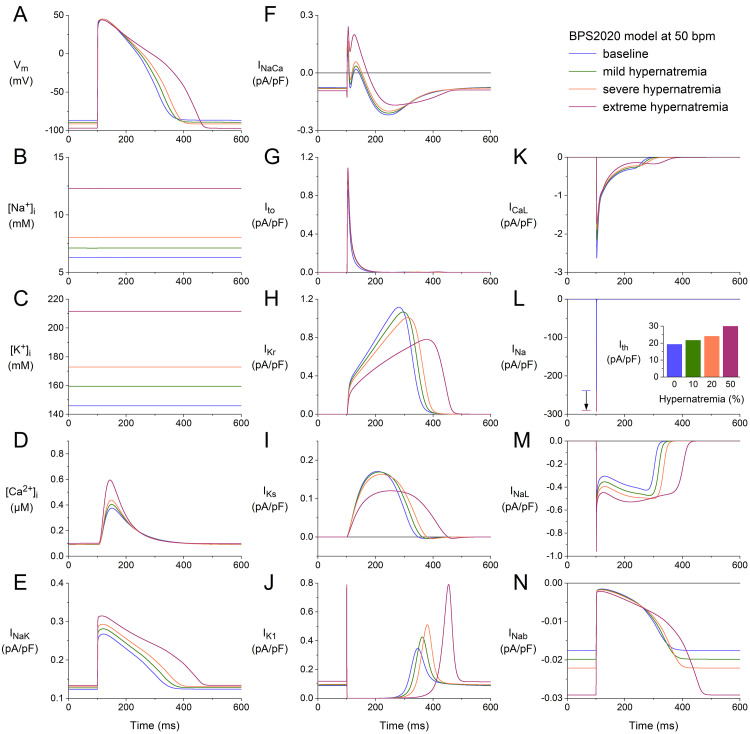
**Effects of hypernatremia on the electrical activity of a single human ventricular cardiomyocyte (BPS2020 model) at a beating rate of 50 bpm.** (A) Membrane potential ($V_{\text{m}}$). (B) Intracellular ${\mathrm{Na}}^{+}$ concentration ([${\mathrm{Na}}^{+}$]_i_). (C) Intracellular $K^{+}$ concentration ([$K^{+}$]_i_). (D) Intracellular ${\mathrm{Ca}}^{\text{2+}}$ concentration ([${\mathrm{Ca}}^{\text{2+}}$]_i_). (E) ${\mathrm{Na}}^{+}$–$K^{+}$ pump current ($I_{\text{NaK}}$). (F) ${\mathrm{Na}}^{+}$–${\mathrm{Ca}}^{\text{2+}}$ exchange current ($I_{\text{NaCa}}$). (G) Transient outward $K^{+}$ current ($I_{\text{to}}$). (H) Rapid delayed rectifier $K^{+}$ current ($I_{\text{Kr}}$). (I) Slow delayed rectifier $K^{+}$ current ($I_{\text{Ks}}$). (J) Inward rectifier $K^{+}$ current ($I_{\text{K1}}$). (K) L-type ${\mathrm{Ca}}^{\text{2+}}$current ($I_{\text{CaL}}$). (L) Fast ${\mathrm{Na}}^{+}$ current ($I_{\text{Na}}$). The vertical arrow indicates the increase in $I_{\text{Na}}$ amplitude with increasing levels of hypernatremia. (M) Late ${\mathrm{Na}}^{+}$ current ($I_{\text{NaL}}$). (N) Background ${\mathrm{Na}}^{+}$ current ($I_{\text{Nab}}$). Note the differences in current scales. The inset to (L) shows the threshold stimulus current ($I_{\text{th}}$) at the different levels of hypernatremia. BPS2020, Bartolucci-Passini-Severi model as published in 2020; bpm, beats per minute.

Fig. 1A shows APs (membrane potential, $V_{\text{m}}$) obtained after a simulated period of 10 min of stimulation at 50 $\min^{-1}$ under control conditions (‘baseline’, blue trace) and at 10, 20, and 50% hypernatremia (green, orange, and purple traces, respectively). Such a 10-minute period is sufficient to obtain steady-state or quasi-steady-state behavior at each level of hypernatremia and each stimulation rate tested. Fig. 1B–D shows the associated intracellular ${\mathrm{Na}}^{+}$, $K^{+}$, and ${\mathrm{Ca}}^{\text{2+}}$ concentrations (denoted by [${\mathrm{Na}}^{+}$]_i_, [$K^{+}$]_i_, and [${\mathrm{Ca}}^{\text{2+}}$]_i_, respectively). The cell shrinkage that is caused by the hyperosmosis of the extracellular solution results in increased levels of both [${\mathrm{Na}}^{+}$]_i_ and [$K^{+}$]_i_ (Fig. 1B,C). The diastolic resting level of [${\mathrm{Ca}}^{\text{2+}}$]_i_ is hardly affected by the hypernatremia, but there is an increase in its systolic peak value with increasing levels of hypernatremia (Fig. 1D).

The activity of the ${\mathrm{Na}}^{+}$–$K^{+}$ pump is, on the one hand, enhanced by the increase in [${\mathrm{Na}}^{+}$]_i_, but on the other hand, reduced by the increase in [$K^{+}$]_i_ as well as by the cell shrinkage* per se* (as observed experimentally, as described in the Introduction section, and represented in the model as set out in the Materials and Methods section). The net effect is an increase in $I_{\text{NaK}}$ (Fig. 1E), which is, however, insufficient to keep [${\mathrm{Na}}^{+}$]_i_ at its baseline level. The activity of the ${\mathrm{Na}}^{+}$–${\mathrm{Ca}}^{\text{2+}}$ exchanger is not only enhanced by the increase in [${\mathrm{Ca}}^{\text{2+}}$]_i_ but also by the cell shrinkage *per se* (as observed experimentally, as described in the Introduction section, and represented in the model as set out in the Materials and Methods section). The net effect is an increase in $I_{\text{NaCa}}$ (Fig. 1F). The time course of the transient outward current ($I_{\text{to}}$; Fig. 1G), which is a $K^{+}$ current, is only slightly dependent on the level of hypernatremia. This is because the activation of $I_{\text{to}}$ is largely determined by the AP upstroke and early repolarization phases, which do not show a marked change (Fig. 1A). Its amplitude increases with increasing hypernatremia, among other things, due to the increase in its driving force by the hyperpolarization of the $K^{+}$ equilibrium potential ($E_{\text{K}}$) as a result of the increase in [$K^{+}$]_i_ (Fig. 1C). The driving force of $I_{\text{Kr}}$ and $I_{\text{Ks}}$ is also increased. Yet, both currents show a decreased amplitude with increasing hypernatremia (Fig. 1H,I). This is largely due to the cell shrinkage-induced decrease in their fully activated conductance (as observed experimentally, as described in the Introduction section, and represented in the model as set out in the Materials and Methods section). $I_{\text{K1}}$ increases with increasing hypernatremia (Fig. 1J), entirely due to its voltage dependence and the hypernatremia-induced hyperpolarization of $V_{\text{m}}$ (Fig. 1A).

$I_{\text{CaL}}$ shows a complex dependence on intracellular and extracellular ion concentrations and voltage. The net effect of the hypernatremia is a decrease in its amplitude (Fig. 1K). The fast ${\mathrm{Na}}^{+}$ current ($I_{\text{Na}}$), on the other hand, shows an increase with increasing hypernatremia (Fig. 1L), which is largely due to the reduction in its steady-state inactivation due to the hyperpolarization of the resting membrane potential between consecutive APs (Fig. 1A). This increase *per se* would result in a faster activation of neighboring cells and an associated increase in conduction velocity. However, this is counteracted by reduced excitability, as reflected by the increase in threshold stimulus current ($I_{\text{th}}$; Fig. 1L, inset). The late $I_{\text{Na}}$ ($I_{\text{NaL}}$) also shows an increase with increasing hypernatremia (Fig. 1M), which is also largely due to the reduction of its steady-state inactivation due to the hyperpolarization of the resting membrane potential between consecutive APs (Fig. 1A). The model cell has several other inward and outward currents, in addition to those shown in Fig. 1E–M. These include the background ${\mathrm{Na}}^{+}$, $K^{+}$, and ${\mathrm{Ca}}^{\text{2+}}$ currents ($I_{\text{Nab}}$, $I_{\text{Kb}}$, and $I_{\text{Cab}}$, respectively) and the sarcolemmal ${\mathrm{Ca}}^{\text{2+}}$ pump current ($I_{\text{pCa}}$). As illustrated in Fig. 1N for $I_{\text{Nab}}$, these other currents also depend on the level of hypernatremia through their dependence on ion concentrations and voltage. However, as illustrated in Fig. 1N, these currents are so small that they hardly contribute to the net membrane current.

The net effect of the hypernatremia-induced changes in ion concentrations (Fig. 1B–D) and membrane currents (Fig. 1E–N) is hyperpolarization and prolongation of the AP (Fig. 1A). The hyperpolarization amounts to 2.5, 4.8, and 10.3 mV under conditions of mild, severe, and extreme hypernatremia, respectively, whereas the AP duration (APD) at 90% repolarization (${\mathrm{APD}}_{\text{90}}$) is increased by 7, 14, and 46%, respectively. As a direct effect of the increase in $I_{\text{Na}}$ amplitude (Fig. 1L), the maximum AP upstroke velocity ((${\mathrm{dV}}_{\text{m}}$/dt)_max_) is increased by 11, 18, and 23% under conditions of mild, severe, and extreme hypernatremia, respectively. At the same time, $I_{\text{th}}$ is increased by 12, 25, and 55%, respectively (Fig. 1L, inset).

**Fig. 2.**
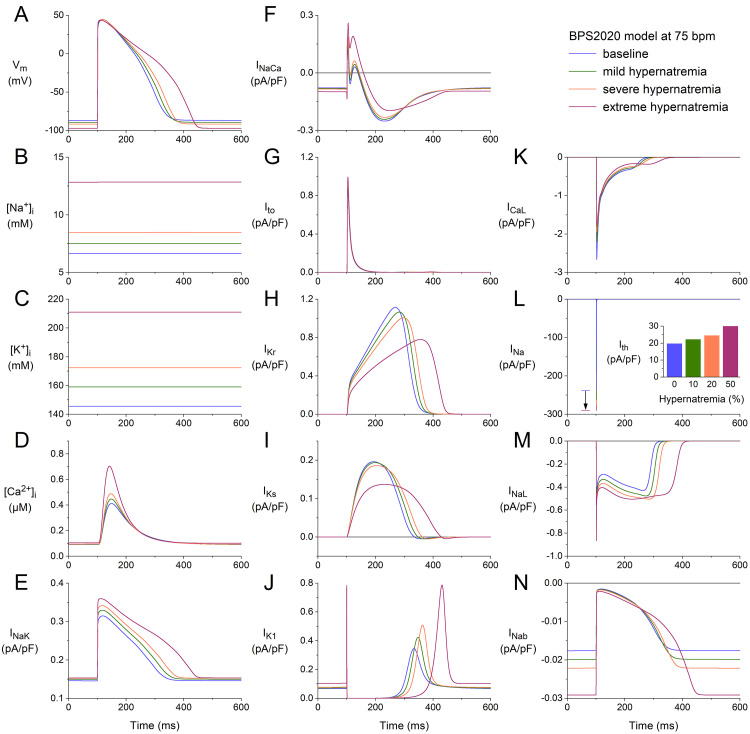
**Effects of hypernatremia on the electrical activity of a single human ventricular cardiomyocyte (BPS2020 model) at a beating rate of 75 bpm.** (A) $V_{\text{m}}$. (B) [${\mathrm{Na}}^{+}$]_i_. (C) [$K^{+}$]_i_. (D) [${\mathrm{Ca}}^{\text{2+}}$]_i_. (E) $I_{\text{NaK}}$. (F) $I_{\text{NaCa}}$. (G) $I_{\text{to}}$. (H) $I_{\text{Kr}}$. (I) $I_{\text{Ks}}$. (J) $I_{\text{K1}}$. (K) $I_{\text{CaL}}$. (L) $I_{\text{Na}}$. The vertical arrow indicates the increase in $I_{\text{Na}}$ amplitude with increasing levels of hypernatremia. (M) $I_{\text{NaL}}$. (N) $I_{\text{Nab}}$. Axis scales are identical to those in Fig. 1. The inset to (L) shows $I_{\text{th}}$ at the different levels of hypernatremia. BPS2020, Bartolucci-Passini-Severi model as published in 2020; bpm, beats per minute.

**Fig. 3.**
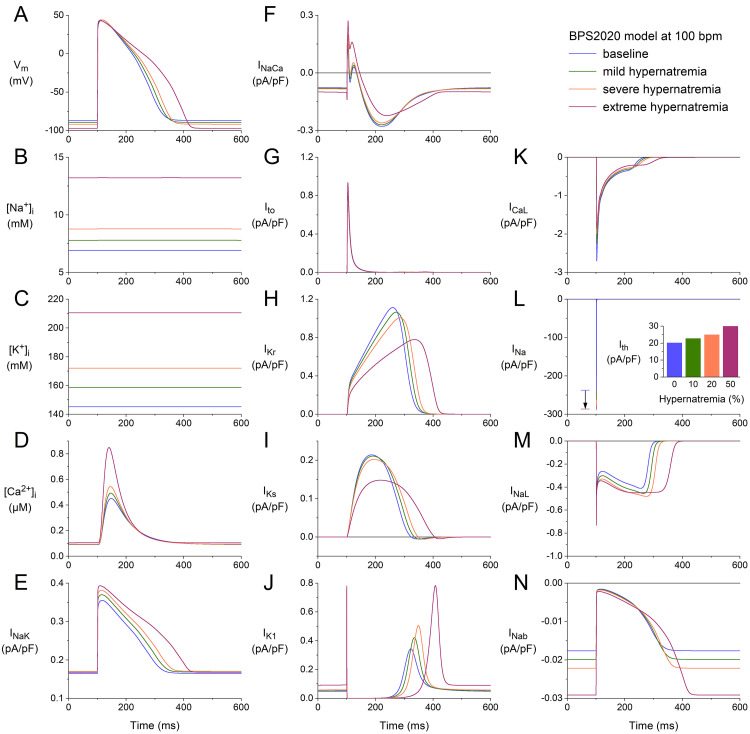
**Effects of hypernatremia on the electrical activity of a single human ventricular cardiomyocyte (BPS2020 model) at a beating rate of 100 bpm.** (A) $V_{\text{m}}$. (B) [${\mathrm{Na}}^{+}$]_i_. (C) [$K^{+}$]_i_. (D) [${\mathrm{Ca}}^{\text{2+}}$]_i_. (E) $I_{\text{NaK}}$. (F) $I_{\text{NaCa}}$. (G) $I_{\text{to}}$. (H) $I_{\text{Kr}}$. (I) $I_{\text{Ks}}$. (J) $I_{\text{K1}}$. (K) $I_{\text{CaL}}$. (L) $I_{\text{Na}}$. The vertical arrow indicates the increase in $I_{\text{Na}}$ amplitude with increasing levels of hypernatremia. (M) $I_{\text{NaL}}$. (N) $I_{\text{Nab}}$. Axis scales are identical to those in Figs. 1,2. The inset to (L) shows $I_{\text{th}}$ at the different levels of hypernatremia. BPS2020, Bartolucci-Passini-Severi model as published in 2020; bpm, beats per minute.

We repeated our simulations with the BPS2020 model at higher stimulation rates of 75 and 100 $\min^{-1}$. The results obtained at these two rates (Figs. 2,3) are qualitatively similar to those obtained at 50 $\min^{-1}$ (Fig. 1). Rate-dependent quantitative differences with respect to ion concentrations include a higher level of [${\mathrm{Na}}^{+}$]_i_ (Figs. 2B,3B), higher peak amplitude of [${\mathrm{Ca}}^{\text{2+}}$]_i_ (Figs. 2D,3D), and a higher activity of the ${\mathrm{Na}}^{+}$–$K^{+}$ pump (Figs. 2E,3E). Rate-dependent quantitative differences concerning individual membrane currents include a decrease in $I_{\text{to}}$ due to the smaller amount of time available between consecutive APs for its relatively slow recovery from inactivation and an increase in $I_{\text{Ks}}$ due to the smaller amount of time available between consecutive APs for its relatively slow deactivation. The hypernatremia-induced AP hyperpolarization and prolongation observed at 50 bpm (Fig. 1A) were also examined at 75 bpm (Fig. 2A) and 100 bpm (Fig. 3A). The same holds for the hypernatremia-induced increase in $I_{\text{Na}}$ amplitude (Figs. 2L,3L) and the associated increase in (${\mathrm{dV}}_{\text{m}}$/dt)_max_, and the hypernatremia-induced increase in $I_{\text{th}}$ (Fig. 2L, inset; Fig. 3L, inset).

### 3.2 Effects of Hypernatremia in the ToR–ORd Model

As the Introduction mentions, the ToR–ORd and BPS2020 models can be considered major updates of the O’Hara *et al*. [30] “ORd” model. Since the ToR–ORd and BPS2020 models were developed largely independently and along different lines, meaning the simulation results obtained with the two models are not *a priori* highly similar, we repeated the above simulations using the ToR–ORd model. This model has default values for the extracellular ${\mathrm{Na}}^{+}$, $K^{+}$, and ${\mathrm{Ca}}^{\text{2+}}$ concentrations (denoted by [${\mathrm{Na}}^{+}$]_e_, [$K^{+}$]_e_, and [${\mathrm{Ca}}^{\text{2+}}$]_e_, respectively) of 140, 5.0, and 1.8 mmol/L, respectively, as opposed to 144, 5.4, and 2.7 mmol/L, respectively, in the BPS2020 model. Furthermore, unlike the BPS2020 model, it includes a ${\mathrm{Cl}}^{-}$ membrane current ($I_{\text{Cl}}$). However, the intracellular ${\mathrm{Cl}}^{-}$ concentration cannot change dynamically, like [${\mathrm{Na}}^{+}$]_i_, [$K^{+}$]_i_, and [${\mathrm{Ca}}^{\text{2+}}$]_i_ can. The equations describing the time or voltage dependence of individual membrane currents may differ between the two models. Moreover, parameters in these equations, such as the fully activated conductance of a specific current, may vary between the two models so that specific currents can have larger or smaller amplitudes in the ToR–ORd model than in the BPS2020 model and, thus, play a more or less important role in the ToR–ORd than in the BPS2020 model.

**Fig. 4.**
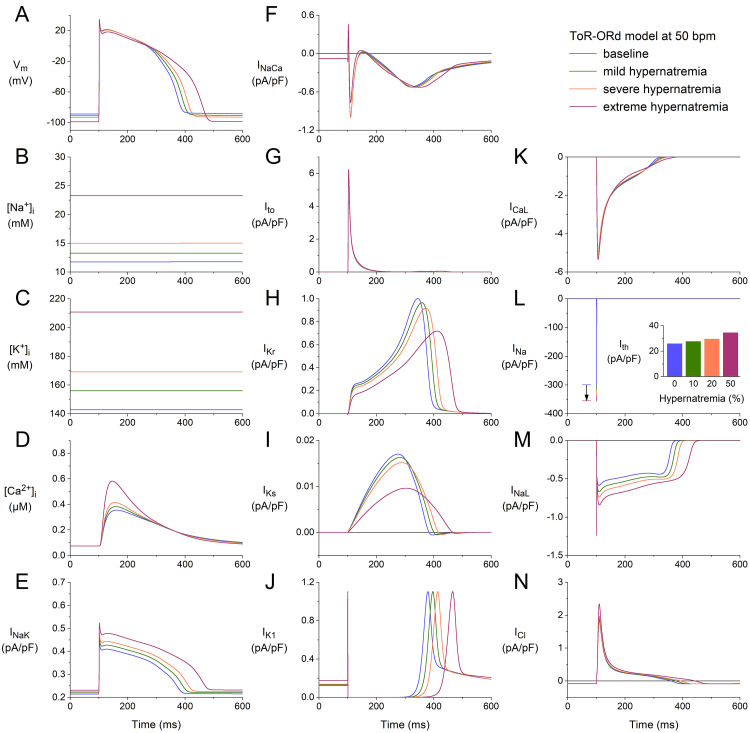
**Effects of hypernatremia on the electrical activity of a single human ventricular cardiomyocyte (ToR–ORd model) at a beating rate of 50 bpm.** (A) $V_{\text{m}}$. (B) [${\mathrm{Na}}^{+}$]_i_. (C) [$K^{+}$]_i_. (D) [${\mathrm{Ca}}^{\text{2+}}$]_i_. (E) $I_{\text{NaK}}$. (F) $I_{\text{NaCa}}$. (G) $I_{\text{to}}$. (H) $I_{\text{Kr}}$. (I) $I_{\text{Ks}}$. (J) $I_{\text{K1}}$. (K) $I_{\text{CaL}}$. (L) $I_{\text{Na}}$. The vertical arrow indicates the increase in $I_{\text{Na}}$ amplitude with increasing levels of hypernatremia. (M) $I_{\text{NaL}}$. (N) Chloride current ($I_{\text{Cl}}$). Note that the ordinate scales are not identical to those in Figs. 1,2,3. The inset to (L) shows $I_{\text{th}}$ at the different levels of hypernatremia. ToR–ORd, Tomek–Rodriguez model following the O’Hara–Rudy dynamic model; bpm, beats per minute.

Fig. 4 shows the results obtained using the ToR–ORd model at a stimulation rate of 50 $\min^{-1}$. The format and time scale are identical to those in Figs. 1,2,3. However, not all of the ordinate scales are identical. Yet, without comparing the ordinate scales, it is immediately clear that the APs from the ToR–ORd and BPS2020 models differ in the presence of a notch. When comparing the ordinate scales of Fig. 4G and Fig. 1G, it is clear that $I_{\text{to}}$ is approximately five times as large in the ToR–ORd model as in the BPS2020 model, giving way to a faster early repolarization and associated AP notch. At the same time, a comparison of the ordinate scales in Fig. 4I and Fig. 1I identifies that $I_{\text{Ks}}$ is almost one order of magnitude smaller in the ToR–ORd model than in the BPS2020 model, which is important for our simulations because $I_{\text{Ks}}$ is one of the currents that is reduced by cell shrinkage *per se*. Other remarkable differences are the [${\mathrm{Na}}^{+}$]_i_ level, which reaches 23.3 mmol/L in the ToR–ORd model vs. 12.3 mmol/L in the BPS2020 model (Fig. 4B vs. Fig. 1B), and the absence of an increase in the amplitude of $I_{\text{K1}}$ with an increase in the level of hypernatremia (Fig. 4J vs. Fig. 1J).

Despite the remarkable differences in [${\mathrm{Na}}^{+}$]_i_ level and some of the individual membrane currents, the net effects of the hypernatremia on the AP configuration are, at least qualitatively, quite similar to those observed using the BPS2020 model. The hypernatremia-induced AP hyperpolarization amounts to 2.3, 4.4, and 10.1 mV under conditions of mild, severe, and extreme hypernatremia, respectively, vs. values of 2.5, 4.8, and 10.3 mV in the BPS2020 model. The ${\mathrm{APD}}_{\text{90}}$ was increased by 6, 12, and 30% under mild, severe, and extreme hypernatremia conditions, whereas this prolongation amounted to 7, 14, and 46%, respectively, in the BPS2020 model. The (${\mathrm{dV}}_{\text{m}}$/dt)_max_ was increased by 7, 11, and 12% under conditions of mild, severe, and extreme hypernatremia, respectively, whereas this increase amounted to 11, 18, and 23%, respectively, in the BPS2020 model. $I_{\text{th}}$ showed an increase of 7, 15, and 34%, respectively, which was 12, 25, and 55%, respectively, in the BPS2020 model.

**Fig. 5.**
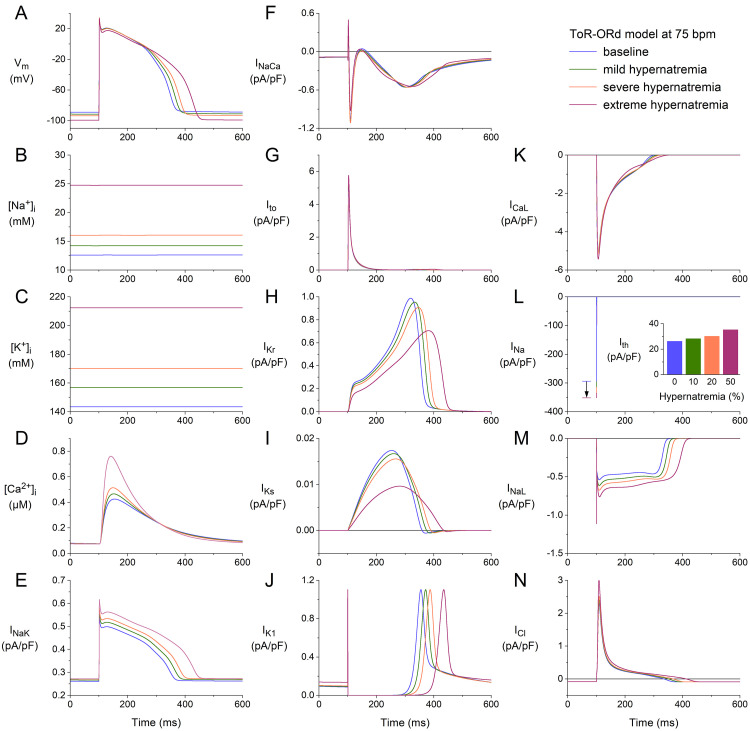
**Effects of hypernatremia on the electrical activity of a single human ventricular cardiomyocyte (ToR–ORd model) at a beating rate of 75 bpm.** (A) $V_{\text{m}}$. (B) [${\mathrm{Na}}^{+}$]_i_. (C) [$K^{+}$]_i_. (D) [${\mathrm{Ca}}^{\text{2+}}$]_i_. (E) $I_{\text{NaK}}$. (F) $I_{\text{NaCa}}$. (G) $I_{\text{to}}$. (H) $I_{\text{Kr}}$. (I) $I_{\text{Ks}}$. (J) $I_{\text{K1}}$. (K) $I_{\text{CaL}}$. (L) $I_{\text{Na}}$. The vertical arrow indicates the increase in $I_{\text{Na}}$ amplitude with increasing levels of hypernatremia. (M) $I_{\text{NaL}}$. (N) $I_{\text{Cl}}$. Axis scales are identical to those in Fig. 4. The inset to (L) shows $I_{\text{th}}$ at the different levels of hypernatremia. ToR–ORd, Tomek–Rodriguez model following the O’Hara–Rudy dynamic model; bpm, beats per minute.

**Fig. 6.**
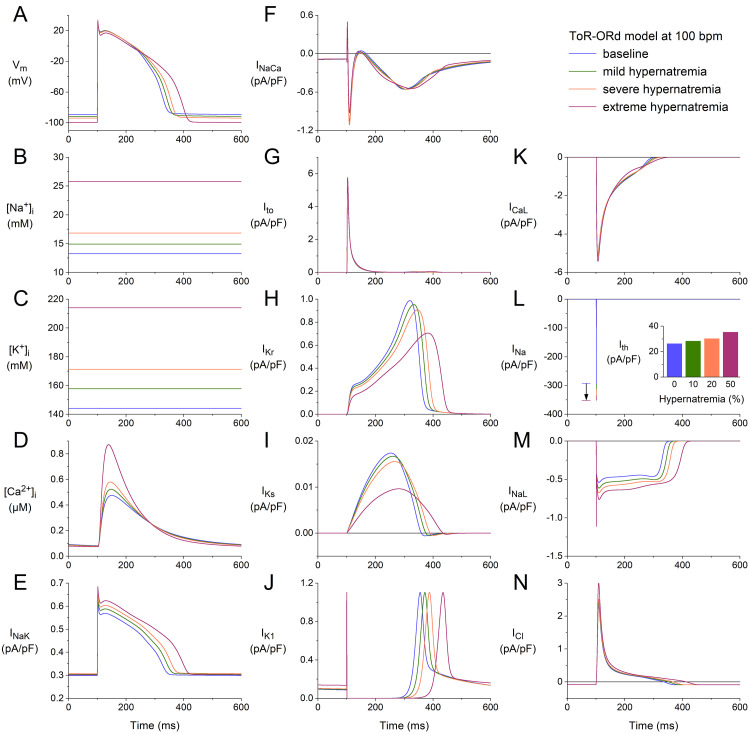
**Effects of hypernatremia on the electrical activity of a single human ventricular cardiomyocyte (ToR–ORd model) at a beating rate of 100 bpm.** (A) $V_{\text{m}}$. (B) [${\mathrm{Na}}^{+}$]_i_. (C) [$K^{+}$]_i_. (D) [${\mathrm{Ca}}^{\text{2+}}$]_i_. (E) $I_{\text{NaK}}$. (F) $I_{\text{NaCa}}$. (G) $I_{\text{to}}$. (H) $I_{\text{Kr}}$. (I) $I_{\text{Ks}}$. (J) $I_{\text{K1}}$. (K) $I_{\text{CaL}}$. (L) $I_{\text{Na}}$. The vertical arrow indicates the increase in $I_{\text{Na}}$ amplitude with increasing levels of hypernatremia. (M) $I_{\text{NaL}}$. (N) $I_{\text{Cl}}$. Axis scales are identical to those in Figs. 4,5. The inset to (L) shows $I_{\text{th}}$ at the different levels of hypernatremia. ToR–ORd, Tomek–Rodriguez model following the O’Hara–Rudy dynamic model; bpm, beats per minute.

We repeated our simulations using the ToR–ORd model at higher stimulation rates of 75 and 100 $\min^{-1}$. The results obtained at these two rates (Figs. 5,6) are qualitatively similar to those obtained at 50 $\min^{-1}$ (Fig. 4). Rate-dependent quantitative differences with respect to ion concentrations include a somewhat higher level of [${\mathrm{Na}}^{+}$]_i_ (Figs. 4B,5B,6B), the substantially higher peak amplitude of [${\mathrm{Ca}}^{\text{2+}}$]_i_ (Figs. 4D,5D,6D), and a somewhat higher activity by the ${\mathrm{Na}}^{+}$–$K^{+}$ pump (Figs. 4E,5E,6E). Rate-dependent quantitative differences with respect to individual membrane currents include a slight decrease in $I_{\text{to}}$, as observed with the BPS2020 model. However, the rate-dependent increase in $I_{\text{Ks}}$ is now only marginal (Figs. 4I,5I,6I). As mentioned, there is no increase in $I_{\text{K1}}$ with increasing hypernatremia (Figs. 4J,5J,6J), in contrast to the BPS2020 model. This is due to differences in the current-voltage relationship of this current between the two models. The hypernatremia-induced AP hyperpolarization and prolongation observed at 50 bpm (Fig. 4A) were also examined at 75 bpm (Fig. 5A) and 100 bpm (Fig. 6A). The same holds for the hypernatremia-induced increase in $I_{\text{Na}}$ amplitude (Figs. 5L,6L) and the associated increase in (${\mathrm{dV}}_{\text{m}}$/dt)_max_, and the hypernatremia-induced increase in $I_{\text{th}}$ (Fig. 5L, inset; Fig. 6L, inset).

### 3.3 Summary of the Effects of Hypernatremia

**Fig. 7.**
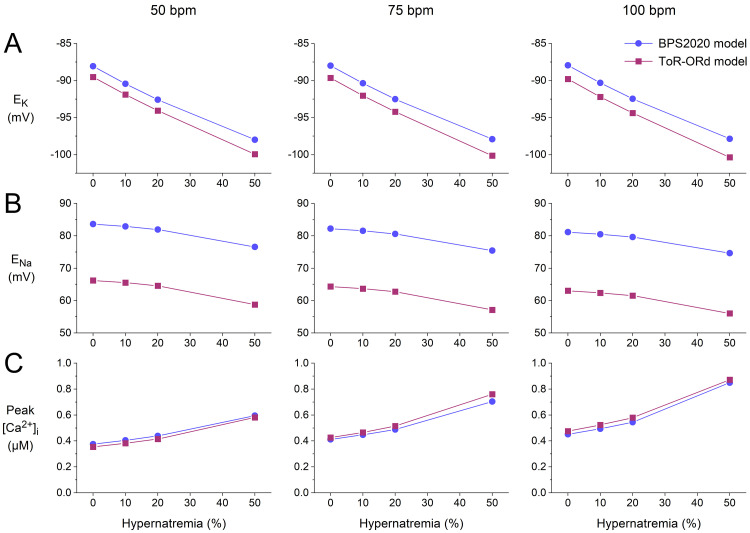
**Effects of hypernatremia on the sodium and potassium equilibrium potentials and the peak intracellular calcium concentration in the BPS2020 and ToR–ORd models of a single human ventricular cardiomyocyte.** (A) $K^{+}$ equilibrium potential ($E_{\text{K}}$) as a function of hypernatremia at stimulation rates of, from left to right, 50, 75, and 100 $\min^{-1}$. (B) ${\mathrm{Na}}^{+}$ equilibrium potential ($E_{\text{Na}}$) as a function of hypernatremia at 50, 75, and 100 $\min^{-1}$ stimulation rates. (C) The peak amplitude of [${\mathrm{Ca}}^{\text{2+}}$]_i_ as a function of hypernatremia at 50, 75, and 100 $\min^{-1}$ stimulation rates. Data from the BPS2020 and ToR–ORd models are shown by filled blue circles and filled purple squares, respectively. ToR–ORd, Tomek–Rodriguez model following the O’Hara–Rudy dynamic model; BPS2020, Bartolucci-Passini-Severi model as published in 2020; bpm, beats per minute.

**Fig. 8.**
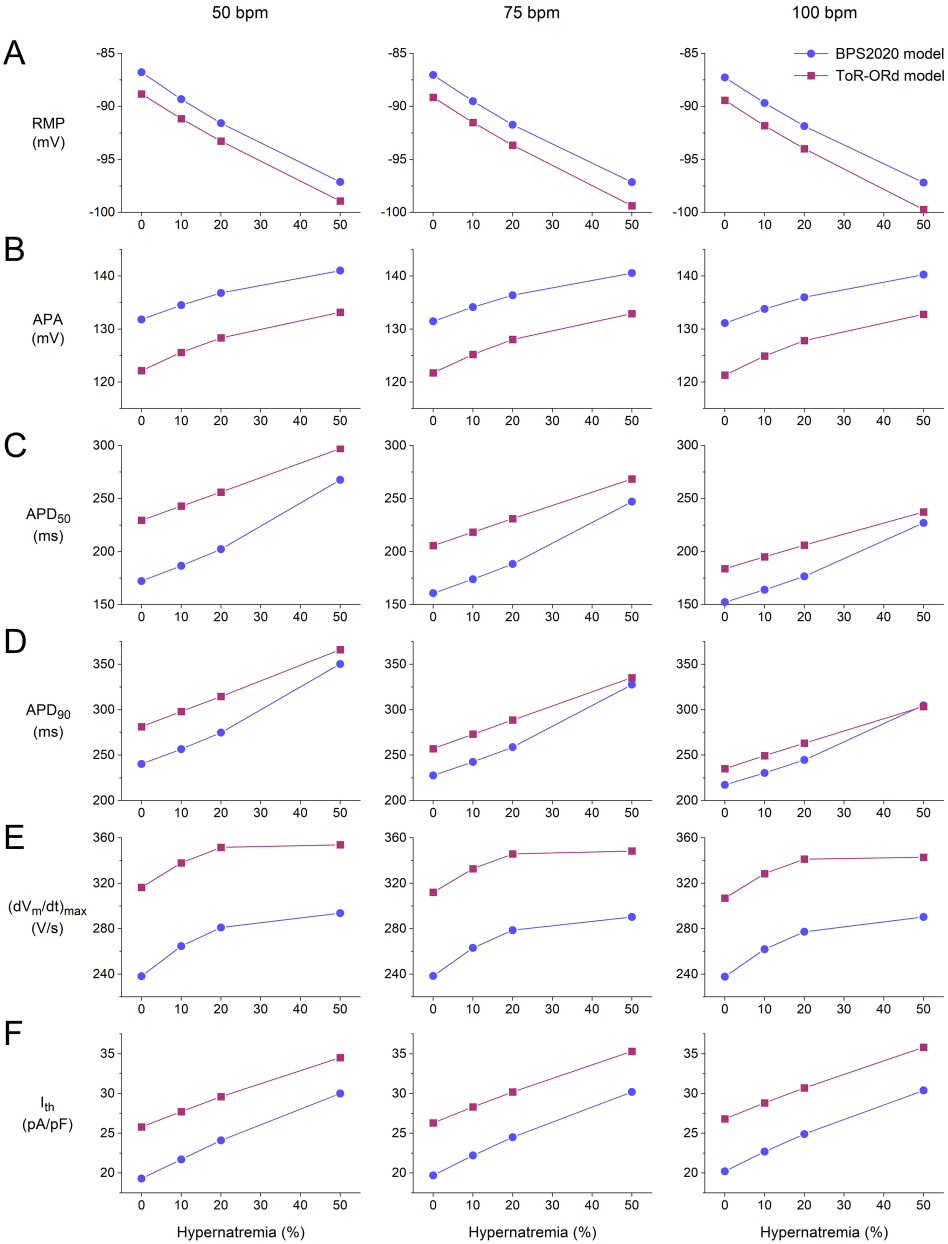
**Action potential (AP) parameters and threshold stimulus current of the BPS2020 and ToR–ORd models of a single human ventricular cardiomyocyte.** (A) Resting membrane potential (RMP) as a function of hypernatremia at left to right, 50, 75, and 100 $\min^{-1}$ stimulation rates. (B) AP amplitude (APA) as a function of hypernatremia at stimulation rates of 50, 75, and 100 $\min^{-1}$. (C) AP duration at 50% repolarization (${\mathrm{APD}}_{\text{50}}$) as a function of hypernatremia at stimulation rates of 50, 75, and 100 $\min^{-1}$. (D) AP duration at 90% repolarization (${\mathrm{APD}}_{\text{90}}$) as a function of hypernatremia at stimulation rates of 50, 75, and 100 $\min^{-1}$. (E) Maximum AP upstroke velocity ((${\mathrm{dV}}_{\text{m}}$/dt)_max_) as a function of hypernatremia at stimulation rates of 50, 75, and 100 $\min^{-1}$. (F) $I_{\text{th}}$ as a function of hypernatremia at stimulation rates of 50, 75, and 100 $\min^{-1}$. Data from the BPS2020 and ToR–ORd models are shown by filled blue circles and filled purple squares, respectively. ToR–ORd, Tomek–Rodriguez model following the O’Hara–Rudy dynamic model; BPS2020, Bartolucci-Passini-Severi model as published in 2020; bpm, beats per minute.

The effects of hypernatremia in the two models of a single human ventricular cardiomyocyte are summarized in Figs. 7,8.

In both models, the $K^{+}$ and ${\mathrm{Na}}^{+}$ equilibrium potentials ($E_{\text{K}}$ and $E_{\text{Na}}$, respectively), as computed from the extracellular and intracellular $K^{+}$ and ${\mathrm{Na}}^{+}$ concentrations, show a hyperpolarization, with a similar dependence on the level of hypernatremia at each of the stimulation rates tested (Fig. 7A,B). The hyperpolarization of $E_{\text{K}}$ underlies the hypernatremia-induced hyperpolarization of the RMP in the two models. The hyperpolarization of $E_{\text{K}}$ and $E_{\text{Na}}$ is associated with changes in the driving force of individual membrane currents, which should be considered when studying the effects of hypernatremia on these currents, as performed in our simulations. Both models show a substantial increase in the peak amplitude of [${\mathrm{Ca}}^{\text{2+}}$]_i_ with increasing levels of hypernatremia (Fig. 7C). At each of the beating rates tested, the two models show a highly similar dependence of this [${\mathrm{Ca}}^{\text{2+}}$]_i_ peak amplitude on the level of hypernatremia.

Fig. 8 shows how the AP parameters and $I_{\text{th}}$ of the BPS2020 and ToR–ORd model cardiomyocytes depend on the level of hypernatremia at each of the stimulation rates tested. As already noted, the resting membrane potential (RMP) shows a hyperpolarization that increases with increasing hypernatremia (Fig. 8A). This hyperpolarization is largely responsible for the observed increase in AP amplitude (APA; Fig. 8B). The hypernatremia-induced AP prolongation does not only translate into a hypernatremia-dependent increase in ${\mathrm{APD}}_{\text{90}}$ (Fig. 8D), as already noted in Sections 3.1 and 3.2, but also into a hypernatremia-dependent increase in the APD at 50% repolarization (${\mathrm{APD}}_{50}$; Fig. 8C).

Fig. 8E demonstrates how (${\mathrm{dV}}_{\text{m}}$/dt)_max_ increases with increasing hypernatremia, reflecting the hypernatremia-induced increase in $I_{\text{Na}}$ amplitude. This increase was only small between 20 and 50% of the hypernatremia, particularly in the case of the ToR–ORd model, whereas $I_{\text{th}}$ shows an almost linear dependence on the level of hypernatremia over the entire range of the hypernatremia tested. Consequently, AP conduction will be impaired at 50% hypernatremia as compared to lower levels of hypernatremia, provided that this high level of hypernatremia does not affect AP conduction in other ways, e.g., by cell shrinkage-induced structural perturbations of the nanodomains at the intercalated disks which are involved in cardiac conduction, and because of the localization of ${\mathrm{Na}}^{+}$ channels in the intercalated disks [35, 36].

### 3.4 Effects of Scaling $I_{\text{CaL}}$

As noted in the Introduction, there is an apparent discrepancy in the experimental data on the effects of acute exposure to a hyperosmotic solution on $I_{\text{CaL}}$ (Table 1). Therefore, we repeated some of our simulations using $I_{\text{CaL}}$ scaling factors other than the factor of 1.00, which was used in the simulations presented in Figs. 1,2,3,4,5,6,7,8. We selected the extreme case of 50% hypernatremia, where the effects are the most pronounced, and simulated both a decrease in $I_{\text{CaL}}$ with a scaling factor of 0.72 and an increase with a scaling factor of 1.45. These scaling factors were derived from the experimental data of Ogura *et al*. [20] and Luo *et al*. [27], respectively (Table 1), disregarding our argument in the Introduction that the decrease in $I_{\text{CaL}}$ amplitude in the study of Ogura *et al*. [20] was due to a rapid increase in [${\mathrm{Ca}}^{\text{2+}}$]_i_ rather than a direct functional effect of the hyperosmosis on the $I_{\text{CaL}}$ channels.

**Fig. 9.**
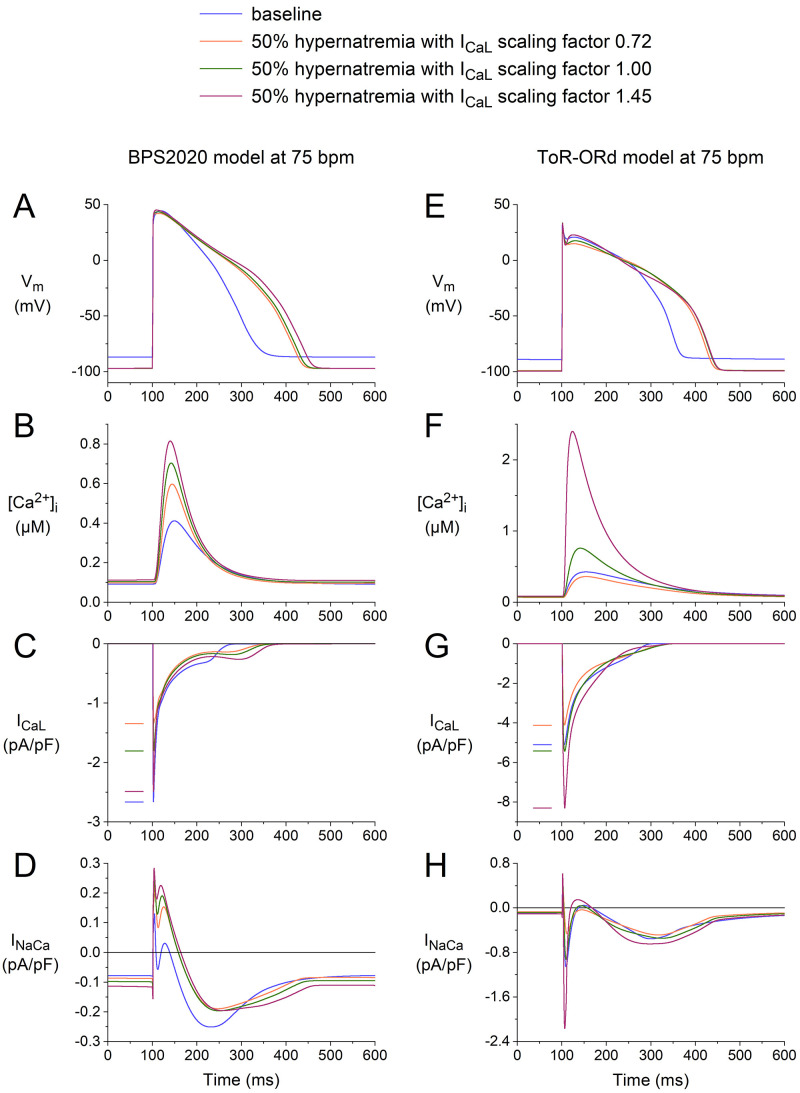
**Effects of 50% hypernatremia and different scaling factors for $I_{\text{Cal}}$ on the electrical activity of the BPS2020 and ToR–ORd models for a single human ventricular cardiomyocyte at a beating rate of 75 bpm.** (A) $V_{\text{m}}$, (B) [${\mathrm{Ca}}^{\text{2+}}$]_i_, (C) $I_{\text{CaL}}$, and (D) $I_{\text{NaCa}}$ in the BPS2020 model. (E) $V_{\text{m}}$, (F) [${\mathrm{Ca}}^{\text{2+}}$]_i_, (G) $I_{\text{CaL}}$, and (H) $I_{\text{NaCa}}$ in the ToR–ORd model. Note the differences in the ordinate scales. ToR–ORd, Tomek–Rodriguez model following the O’Hara–Rudy dynamic model; BPS2020, Bartolucci-Passini-Severi model as published in 2020; bpm, beats per minute.

The results of our simulations are shown in Fig. 9, focusing on the effects on $I_{\text{CaL}}$ and the associated effects on [${\mathrm{Ca}}^{\text{2+}}$]_i_ and $I_{\text{NaCa}}$. In both models, increasing the $I_{\text{CaL}}$ scaling factor from 1.00 to 1.45 resulted in an increase in the inward peak of $I_{\text{CaL}}$ (Fig. 9C,G), an increase in peak [${\mathrm{Ca}}^{\text{2+}}$]_i_ (Fig. 9B,F), and an increase in the activity of the ${\mathrm{Na}}^{+}$–${\mathrm{Ca}}^{\text{2+}}$ exchanger (Fig. 9D,H). As might be anticipated, decreasing the $I_{\text{CaL}}$ scaling factor from 1.00 to 0.72 had the opposite effects. Interestingly, decreasing the $I_{\text{CaL}}$ scaling factor from 1.00 to 0.72 still results in a substantial increase in peak [${\mathrm{Ca}}^{\text{2+}}$]_i_ as compared to baseline in the BPS2020 model (Fig. 9B), but not in the ToR–ORd model, where there is a small decrease (Fig. 9F). Thus, there would be a positive effect on the contractile apparatus according to the BPS2020 model and a small negative effect according to the ToR–ORd model. Apparently, the ‘crossover’ from a positive to a negative effect has a different degree of decrease in the $I_{\text{CaL}}$ scaling factor in the two models. In this regard, it should be noted that an increase in [${\mathrm{Ca}}^{\text{2+}}$]_i_ compared to baseline, despite a decrease in the $I_{\text{CaL}}$ scaling factor to 0.72, is largely due to the reduction in cell volume associated with the hypernatremia. Thus, even a substantially reduced amount of ${\mathrm{Ca}}^{\text{2+}}$ ions entering the cell or released from the sarcoplasmic reticulum can still increase [${\mathrm{Ca}}^{\text{2+}}$]_i_, as in the BPS2020 model, or cause only a small decrease, as in the ToR–ORd model.

### 3.5 Effects of Ion Current Scaling vs. Cell Shrinkage

**Fig. 10.**
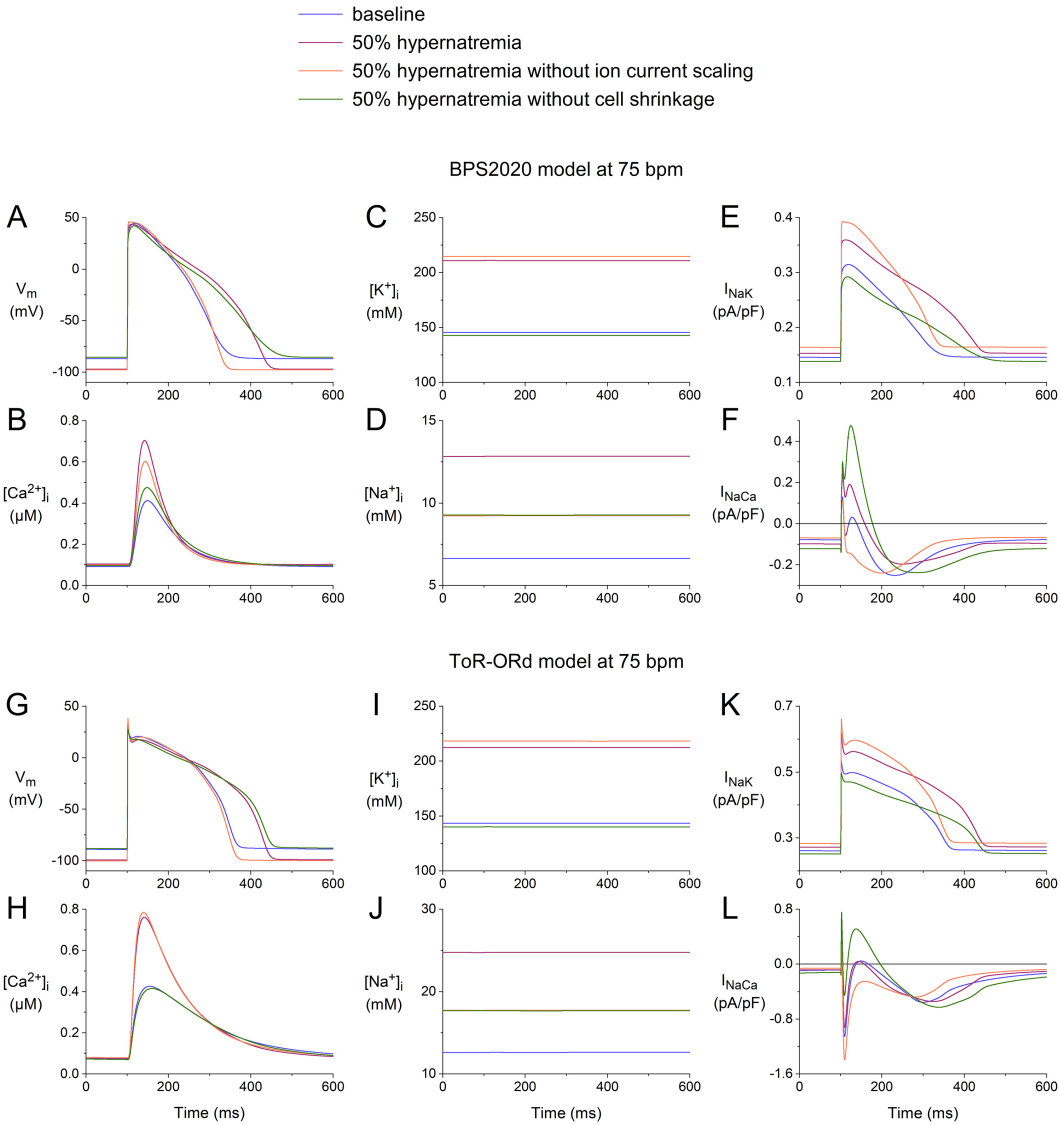
**Effects of 50% hypernatremia in the presence and absence of ion current scaling and the presence and absence of cell shrinkage on the electrical activity of the BPS2020 and ToR–ORd models for a single human ventricular cardiomyocyte at a beating rate of 75 bpm.** (A) $V_{\text{m}}$, (B) [${\mathrm{Ca}}^{\text{2+}}$]_i_, (C) [$K^{+}$]_i_, (D) [${\mathrm{Na}}^{+}$]_i_, (E) $I_{\text{NaK}}$, and (F) $I_{\text{NaCa}}$ in the BPS2020 model. (G) $V_{\text{m}}$, (H) [${\mathrm{Ca}}^{\text{2+}}$]_i_, (I) [$K^{+}$]_i_, (J) [${\mathrm{Na}}^{+}$]_i_, (K) $I_{\text{NaK}}$, and (L) $I_{\text{NaCa}}$ in the ToR–ORd model. Note the differences in the ordinate scales. Note also that the green and orange traces coincidentally overlap almost completely in both (D) and (J). ToR–ORd, Tomek–Rodriguez model following the O’Hara–Rudy dynamic model; BPS2020, Bartolucci-Passini-Severi model as published in 2020; bpm, beats per minute.

When simulating hypernatremia, we incorporated both the hypernatremia-induced cell shrinkage and the hypernatremia-induced changes in $I_{\text{Kr}}$, $I_{\text{Ks}}$, $I_{\text{NaCa}}$, and $I_{\text{NaK}}$ using the scaling factors listed in Table 2. To test the effects of cell shrinkage and ion current scaling *per se*, we simulated two hypothetical cases of hypernatremia, one in the absence of cell shrinkage and one in the absence of ion current scaling. As in the simulations presented in Fig. 9, we selected the extreme 50% level of hypernatremia, where the effects are the most pronounced. The results of our simulations are shown in Fig. 10, focusing on [${\mathrm{Na}}^{+}$]_i_, [$K^{+}$]_i_, and [${\mathrm{Ca}}^{\text{2+}}$]_i_, and the associated $I_{\text{NaK}}$ and $I_{\text{NaCa}}$.

Comparison of the APs obtained under baseline conditions and upon 50% hypernatremia without ion current scaling with those obtained during 50% hypernatremia with and without cell shrinkage (Fig. 10A,G) reveals that the hypernatremia-induced AP prolongation is largely determined by the ion current scaling, which includes scaling factors of 0.6, 0.5, and 0.333 for each of the repolarizing currents $I_{\text{Kr}}$, $I_{\text{Ks}}$, and $I_{\text{NaK}}$, respectively (Table 2). Similarly, a comparison of the intracellular ion concentrations obtained under the different conditions (Fig. 10B–D,H–J) shows that the changes in [${\mathrm{Ca}}^{\text{2+}}$]_i_, [$K^{+}$]_i_, and [${\mathrm{Na}}^{+}$]_i_ are largely, but certainly not completely, determined by the cell shrinkage. In particular, in the case of [${\mathrm{Na}}^{+}$]_i_ (Fig. 10D,J), the ion current scaling has a strong effect. For example, in the ToR–ORd model, the hypernatremia induces an increase in [${\mathrm{Na}}^{+}$]_i_ from its baseline value of 12.6 mmol/L to 24.7 mmol/L in the presence of the ion current scaling, which is a substantially smaller increase to 17.7 mmol/L in the absence of the ion current scaling (Fig. 10J). This effect is not highly surprising, given the hypernatremia-induced decrease in $I_{\text{NaK}}$ and increase in $I_{\text{NaCa}}$ (Table 2), which correspond to a reduced activity of the ${\mathrm{Na}}^{+}$–$K^{+}$ pump and an enhanced activity of the ${\mathrm{Na}}^{+}$–${\mathrm{Ca}}^{\text{2+}}$ exchanger, respectively, both of which tend to increase [${\mathrm{Na}}^{+}$]_i_, in the presence of the ion current scaling. The hypernatremia-induced decrease in $I_{\text{NaK}}$ and increase in $I_{\text{NaCa}}$ are not immediately apparent from a direct comparison of the $I_{\text{NaK}}$ traces in Fig. 10E,K and the $I_{\text{NaCa}}$ traces in Fig. 10F,L. However, it should be noted that $I_{\text{NaK}}$ and $I_{\text{NaCa}}$ depend on the intracellular ion concentrations, which reached different levels under the four conditions tested.

## 4. Discussion

### 4.1 Effects of Acute Hypernatremia on the Cellular Action Potential

Owing to the many effects of acute hypernatremia and the associated cell shrinkage and changes in intracellular ion concentrations on individual membrane currents, it is difficult to predict, if not qualitatively, then at least quantitatively, how acute hypernatremia will affect the ventricular AP. This is where comprehensive computer models of the ventricular cardiomyocyte come into play. With such models, it is possible to determine and understand the effects of different levels of acute hypernatremia on the individual membrane currents and their net effects on the ventricular AP. In the present study, we used two different comprehensive computer models of an isolated human ventricular cardiomyocyte to assess the effects of mild to extreme hypernatremia on the electrophysiology of such a cardiomyocyte. We observed a hyperpolarization of the RMP, a prolongation of the AP, an increase in (${\mathrm{dV}}_{\text{m}}$/dt)_max_, and an increase in $I_{\text{th}}$ at all levels of hypernatremia. The magnitude of these effects increased with increasing levels of hypernatremia.

Experimental data on the cardiac effects of acute hypernatremia at the cellular level are scarce. What we do know from the work of Bou-Abboud and Nattel [37] is that canine Purkinje fibers show small but statistically highly significant increases in their ${\mathrm{APD}}_{50}$ (+12.0%) and ${\mathrm{APD}}_{\text{95}}$ (+5.4%) and in their (${\mathrm{dV}}_{\text{m}}$/dt)_max_ (+4.7%) when [${\mathrm{Na}}^{+}$]_e_ is increased from 141 to 161 mmol/L (14% hypernatremia). Our simulation results at 10–20% hypernatremia, albeit for human ventricular cardiomyocytes rather than canine Purkinje fibers, correlate well with these experimental observations. More experimental data have been obtained on the effects of hyperosmotic extracellular solutions on the AP of cardiac myocytes at the cellular level, although these were obtained with sucrose-induced hyperosmosis rather than hypernatremia. From the 1997 study by Ogura *et al*. [20], we know that guinea pig ventricular cardiomyocytes, when stimulated at 1 Hz, show an increase in ${\mathrm{APD}}_{\text{90}}$ of 10 ± 3% (mean ± SEM, *n* = 4) and 11 ± 2% (*n* = 5) in 20% and 50% hyperosmotic solutions, respectively. In a more recent study, also using guinea pig ventricular cardiomyocytes stimulated at 1 Hz, Ogura *et al*. [22] observed a 16.7 ± 2.4% increase in ${\mathrm{APD}}_{\text{90}}$ (mean ± SEM, *n* = 5) in a 50% hyperosmotic solution as well as a 6.6 ± 0.2 mV hyperpolarization of the RMP. Qualitatively, such an increase in [$K^{+}$]_i_ is supported by the –2.8 ± 0.3 and –7.3 ± 0.7 mV (mean ± SEM, *n* = 9) hyperpolarizing shifts in the $I_{\text{K1}}$ reversal potential for 30% and 80% hyperosmotic solutions [23]. Although obtained with guinea pig rather than human ventricular cardiomyocytes and with a sucrose-induced hyperosmotic extracellular solution rather than a hypernatremic one, these experimental data on AP prolongation and RMP hyperpolarization correlate well with our simulation results. One would expect hyperosmosis and hypoosmosis to have opposite effects on APD, and this is indeed the case. Both Groh *et al*. [38] and Kocic *et al*. [39] found a decrease in ${\mathrm{APD}}_{\text{90}}$ in guinea pig ventricular cardiomyocytes upon hypoosmosis.

Ogura *et al*. [22] attributed the hyperpolarization of the RMP to an increase in [$K^{+}$]_i_ in the osmotically shrunken cardiomyocytes, as also proposed by Missan *et al*. [23]. This is supported by our simulation results, which show an increase in both models. The increase in [$K^{+}$]_i_ is even quantitatively very similar in the two models, which is, however, less the case for the absolute increase in [${\mathrm{Na}}^{+}$]_i_. In this regard, we have to note that there is already a substantial difference in [${\mathrm{Na}}^{+}$]_i_ between the two models at baseline, in contrast to the baseline level of [$K^{+}$]_i_. For example, at 75 bpm, [${\mathrm{Na}}^{+}$]_i_ is 12.6 mmol/L in the ToR–ORd model and 6.6 mmol/L in the BPS2020 model. These values increase to 24.7 and 12.8 mmol/L, respectively, with 50% hypernatremia. Thus, a near doubling of [${\mathrm{Na}}^{+}$]_i_ can be observed in both models.

### 4.2 Experimental Data on the Effects of Acute Hypernatremia on Individual Ion Currents

As mentioned in the Introduction, data on the direct effects of acute hypernatremia on individual membrane currents of cardiac myocytes are lacking. Data on changes in individual membrane currents of single cardiac myocytes that are acutely exposed to hyperosmotic solutions induced by the addition of sucrose or mannitol rather than hypernatremia are not equivocal, particularly in the case of $I_{\text{CaL}}$. This is even more the case when data on hypoosmosis are considered. One would expect hyperosmosis and hypoosmosis to have opposite effects on individual ion currents, yet this is not always the case. From the data summarized in Table 1, one would expect an increase in both $I_{\text{Kr}}$ and $I_{\text{Ks}}$ upon hypoosmosis, while $I_{\text{K1}}$ is unaffected. Groh *et al*. [38] reported no significant changes in $I_{\text{Kr}}$ or $I_{\text{K1}}$ in guinea pig ventricular cardiomyocytes exposed to a 20% hypoosmotic solution, whereas $I_{\text{Ks}}$ increased (as expected) by ≈40%. Kocic *et al*. [39] also observed an increase in $I_{\text{Ks}}$. The application of a 70% hypoosmolar bath solution by Sasaki *et al*. [26] increased $I_{\text{Kr}}$ (as expected), whereas no noticeable change in $I_{\text{K1}}$ was observed, which was in agreement with their observations with a 130% hyperosmolar solution. Furthermore, Rees *et al*. [40] provided evidence that cell swelling enhanced $I_{\text{Ks}}$ (as expected) while inhibiting (rather than increasing) $I_{\text{Kr}}$. Thus, these data on $I_{\text{Ks}}$ and $I_{\text{K1}}$ in response to hypoosmosis are entirely consistent with the data in response to hyperosmosis. However, the data on $I_{\text{Kr}}$ are not: $I_{\text{Kr}}$ is either increased, decreased, or unaffected by hypoosmosis.

From the data summarized in Table 1, one would also expect a decrease in $I_{\text{NaCa}}$ and an increase in $I_{\text{NaK}}$ in response to hypoosmosis. In about half of their cells, Sasaki *et al*. [26] observed a large increase in $I_{\text{NaK}}$ upon superfusion with a hypoosmolar bath solution, consistent with their observations with a 130% hyperosmolar solution. Whalley *et al*. [25] also observed stimulation of the ${\mathrm{Na}}^{+}$–$K^{+}$ pump during exposure to hypoosmolar solutions, consistent with their observations with hyperosmolar solutions. Wright *et al*. [29] showed a decrease in $I_{\text{NaCa}}$ in response to 1.3-fold hypoosmotic treatment, consistent with their observations with hyperosmolar solutions. Thus, all of these data on $I_{\text{NaCa}}$ and $I_{\text{NaK}}$ in response to hypoosmosis are consistent with the data in response to hyperosmosis.

There are also some data on $I_{\text{CaL}}$ in response to hypoosmosis. Groh *et al*. [38] reported no significant changes in $I_{\text{CaL}}$ (measured with 10 mmol/L EGTA in the pipette solution) in guinea pig ventricular cardiomyocytes exposed to a 20% hypoosmotic solution. Similarly, Sasaki *et al*. [26] observed no apparent change in $I_{\text{CaL}}$ (measured with 5.0 mmol/L EGTA or 10 mmol/L BAPTA) upon application of a 70% hypoosmolar bath solution, in agreement with their observations with a 130% hyperosmolar solution. Thus, these data on $I_{\text{CaL}}$ in response to hypoosmosis support the hyperosmosis data of Ogura *et al*. [20] and Sasaki *et al*. [26] rather than those of Luo *et al*. [27].

Unfortunately, experimental data on the effects of hyperosmosis, let alone hypernatremia, on individual membrane currents are limited to the currents listed in Tables 1,2. Most importantly, there are no data on the effects of hyperosmosis on $I_{\text{to}}$ and $I_{\text{Na}}$. If such data had been available, we would have incorporated these into our models. Experimental data on the effects of hyperosmosis on intercellular coupling are also lacking, making conduction studies in strand or tissue models uncertain.

### 4.3 Experimental Data on the Effects of Acute Hypernatremia on the Electrocardiogram (ECG)

There are not many systematic studies of the effects of acute hypernatremia on the ECG. In a study of seven anesthetized dogs, Eliakim *et al*. [41] regularly observed bradycardia, a decrease in P wave amplitude, an increase in QRS complex amplitude, and QT prolongation after intravenous administration of hypertonic saline to induce acute hypernatremia. Gibson *et al*. [42] studied ECG changes in 14 anesthetized dogs upon intravenous infusion of sodium chloride (increase in serum ${\mathrm{Na}}^{+}$ levels of 28–56%, averaging 41%) and consistently observed an increase in the rate-corrected QT interval (3–26%, averaging 11%) and a decrease in the amplitude of the P wave and the QRS complex, without QRS prolongation, and with variable minor changes in rate and negligible changes in PR interval. Importantly, these ECG changes occurred with only a 5–15% increase in serum ${\mathrm{Na}}^{+}$ levels, and only one dog presented an arrhythmia, which was transient and occurred at the start of the infusion. In a study of 20 rabbits subjected to extreme acute hypernatremia, electrocardiographic tracings remained normal until respiratory arrest, “except in a few instances in which peaked T waves were observed in the terminal stages” [43]. Overall, these *in vivo* data from laboratory animals are inconsistent, except perhaps for increased QT prolongation. The inconsistencies may be related, at least in part, to the level of hypernatremia and a reduction in P and QRS amplitude due to alterations in blood conductivity with a short-circuiting effect on myocardial potentials, as discussed by Gibson *et al*. [42], although this cannot fully explain the conflicting data. An increase in QT prolongation is consistent not only with the increase in APD that was observed *in vitro* (see Section 4.1) and that we observed *in silico* (Fig. 8C,D) but also with clinical and experimental ECG data in case reports of the effects of (often extreme) acute hypernatremia, including a prolonged rate-corrected Q-U interval in a 12-year-old girl [44], QT prolongation in an 11-year-old girl [9], and QT prolongation in a 29-year-old woman [8].

### 4.4 Intracellular ${\mathrm{Ca}}^{\text{2+}}$ Concentration And Contractile (dys)function

Hypernatremia may also lead to left ventricular (LV) contractile dysfunction. In a prospective cohort study of subarachnoid hemorrhage patients, Fisher *et al*. [6] observed that hypernatremia was an independent predictor of a reduced LV ejection fraction of <50%. In line with this observation, King *et al*. [45] demonstrated that elevating [${\mathrm{Na}}^{+}$]_e_ from 145 to 155 mmol/L in Langendorff-perfused isolated rat heart preparations decreased LV developed pressure (LVdP). Fisher *et al*. [6] hypothesized that the elevated [${\mathrm{Na}}^{+}$]_e_ causes more ${\mathrm{Ca}}^{\text{2+}}$ to exit the cell via the sarcolemmal ${\mathrm{Na}}^{+}$–${\mathrm{Ca}}^{\text{2+}}$ exchanger, which then results in reduced levels of [${\mathrm{Ca}}^{\text{2+}}$]_i_ available for cardiac myocyte contraction, thus causing a negative inotropic effect. On the other hand, hypernatremia ranging from 163 to 218 mmol/L in 10 anesthetized dogs resulted in statistically significant increases in cardiac output, heart rate, and maximum rate of rise in LVdP in the study by Goodyer *et al*. [46].

The increase in the systolic peak amplitude of [${\mathrm{Ca}}^{\text{2+}}$]_i_ that we found in our simulations is consistent with the findings of Goodyer *et al*. [46], especially when we compare the peak amplitude of [${\mathrm{Ca}}^{\text{2+}}$]_i_ obtained at 20–50% hypernatremia and a beating rate of 75–100 bpm with the baseline peak [${\mathrm{Ca}}^{\text{2+}}$]_i_ at 50 bpm to account for the increase in heart rate that accompanies the hypernatremia. At 10% hypernatremia, which best reflects mild hypernatremia, our simulations still predict an increase in peak [${\mathrm{Ca}}^{\text{2+}}$]_i_, although less pronounced, which seems to contradict the observations of Fisher *et al*. [6] and King *et al*. [45] that mild hypernatremia impairs LVdP. This may indicate a shortcoming in our models; however, it may also point to a negative effect on the contractile apparatus that requires a higher increase in peak [${\mathrm{Ca}}^{\text{2+}}$]_i_, as obtained at 20–50% hypernatremia, to be compensated. Notably, the hypothesis of Fisher *et al*. [6] regarding a hypernatremia-induced reduction in the level of [${\mathrm{Ca}}^{\text{2+}}$]_i_ available for cardiac myocyte contraction is not supported by our simulations.

In our simulations, there is an increase in peak [${\mathrm{Ca}}^{\text{2+}}$]_i_ at all levels of hypernatremia, yet it is most prominent at 50% hypernatremia. For example, in the BPS2020 model, with a beating rate of 75 bpm, the peak [${\mathrm{Ca}}^{\text{2+}}$]_i_ is 0.704 µmol/L at 50% hypernatremia compared to 0.412 µmol/L at baseline (+71%; Fig. 2D). Yet, the associated changes in $I_{\text{CaL}}$ and $I_{\text{NaCa}}$ do not seem to be large enough to explain this increase in [${\mathrm{Ca}}^{\text{2+}}$]_i_ fully. Early in the AP, there is an increase in reverse mode $I_{\text{NaCa}}$, making more ${\mathrm{Ca}}^{\text{2+}}$ ions enter the cell, but on the other hand, there is a decrease in peak $I_{\text{CaL}}$ (Fig. 2F,K). In this regard, it should be noted that the increase in [${\mathrm{Ca}}^{\text{2+}}$]_i_ with increasing levels of hypernatremia is largely due to the associated decrease in cell volume. The free ${\mathrm{Ca}}^{\text{2+}}$ ions reside in a 32% smaller myoplasmic volume compared to baseline, which *per se* results in a 47% increase in [${\mathrm{Ca}}^{\text{2+}}$]_i_. Thus, even a reduced amount of ${\mathrm{Ca}}^{\text{2+}}$ ions entering the cell or released from the sarcoplasmic reticulum can still increase [${\mathrm{Ca}}^{\text{2+}}$]_i_.

### 4.5 Clinical Settings

It should be noted that in all of the above, the focus is on acute hypernatremia, with the associated abrupt cell shrinkage; however, the more common hypernatremia in the clinic is a more gradually developing and chronic hypernatremia. In this context, the changes in intracellular volume and osmolarity and those in individual membrane currents will be much smaller. It is likely that, as a consequence, the effects of this type of hypernatremia will be much less pronounced than in our simulations. Unfortunately, there are no cellular electrophysiological data on this type of hypernatremia to support this hypothesis. Furthermore, in the clinical management of the more gradually developing hypernatremia, it is important to consider not only the absolute level of the serum ${\mathrm{Na}}^{+}$ concentration but also the time of development of the hypernatremia because too slow or too rapid corrections of the hypernatremia are both associated with a poor patient prognosis [47, 48].

Another point of attention is the extreme 50% hypernatremia we used in our simulations. Such a high level of hypernatremia is clinically limited to a few cases reported worldwide [10, 11, 12, 13, 14, 15] and some of the cases in the commonly referenced study by Finberg *et al*. [49], in which they describe the dramatic mix-up of salt and sugar in the preparation of feedings that were received by 14 hospitalized infants, six of whom died. The highest level of ${\mathrm{Na}}^{+}$ observed in the latter study was as high as 274 mmol/L (with a non-fatal outcome).

### 4.6 Limitations

Our computer simulations were performed using the default versions of the ToR–ORd and BPS2020 models. It should be noted that both models represent endocardial cardiomyocytes with their default settings. However, epicardial and mid-myocardial versions of each model are also available. In both models, changing the cell type affects the kinetics and the amplitude of $I_{\text{to}}$, the amplitude of $I_{\text{CaL}}$, $I_{\text{Kb}}$, $I_{\text{Kr}}$, $I_{\text{Ks}}$, $I_{\text{K1}}$, $I_{\text{NaCa}}$, $I_{\text{NaK}}$, and $I_{\text{NaL}}$, and parameters related to the uptake and release of ${\mathrm{Ca}}^{\text{2+}}$ ions by the sarcoplasmic reticulum. Given the highly similar simulation data obtained using the two models, despite differences in the amplitude and kinetics of individual ion currents, it is highly unlikely that substantially different simulation results will be obtained when repeating our simulations with the epicardial or mid-myocardial versions of each model.

It should be recognized that experimental data on the effects of acute exposure to a hyperosmotic solution on individual cardiac ion currents are scarce and obtained at different levels of hyperosmolarity (Table 1), so we had to estimate several of the values for use in our simulations (Table 2). We did this using linear interpolation and extrapolation, which we considered the best possible option. However, there is no evidence that the observed changes in individual ion currents are actually linearly dependent on the level of hyperosmolarity over the range studied.

One might anticipate cell shrinkage to affect the membrane capacitance. However, Ogura *et al*. [22] carried out specific experiments on this issue in which the membrane capacitance of seven guinea pig ventricular cardiomyocytes was monitored during sequential superfusion with control, 50% hyperosmotic, and 50% hypoosmotic solutions (varying sucrose levels). The superfusion with the anisosmotic solutions caused cell shrinkage and swelling but did not affect the membrane capacitance. Based on the experimental data of Ogura *et al*. [22], we did not change the membrane capacitance in our simulations of hypernatremia. However, it may well be that human ventricular cardiomyocytes show a change in membrane capacitance upon acute hypernatremia that we have not accounted for in our simulations.

It should be noted that our study is limited to the electrophysiological effects of acute hypernatremia on a single ventricular cardiomyocyte (for which experimental data on the effects of the hyperosmolarity are available, as summarized in Table 1). As such, it is difficult to predict the effects of acute hypernatremia on the tissue or whole-heart level from our simulations. For that, data are required on the effects of acute hypernatremia on other cell types (sinoatrial, atrial, atrioventricular, Purkinje) and intercellular coupling.

It can be argued that hypernatremia-induced cell shrinkage affects stretch-activated ion channels in the cell membrane, thereby affecting the AP and ion flow across the cell membrane. However, although very comprehensive, the two models we used do not include stretch-activated channels. To overcome this limitation, we should have implemented the various stretch-activated ion channels in the two models and calibrated the resulting extended models to experimental data, as was recently performed for the ToR–ORd model by Buonocunto *et al*. [50].

Both hypoosmotic and hyperosmotic stress can induce the cardiac T-tubules to seal, which could dramatically alter the ${\mathrm{Ca}}^{\text{2+}}$ handling and AP propagation [51], although this was not considered in our study. However, this sealing is a threshold-dependent process, as observed by Uchida *et al*. [52] in a study on isolated mouse left ventricular cardiomyocytes. In the case of hyperosmolarity induced by the addition of extra NaCl to the extracellular Tyrode’s solution, the threshold for the sealing effect is at a hyperosmolarity of ≈65 mOsm/L [52], *i.e.*, at ≈22% hypernatremia. This suggests that our simulation results obtained at hypernatremia levels >20% should be considered with some caution regarding the potential role of T-tubular sealing.

## 5. Conclusions

In the present study, we have used two different comprehensive computer models of an isolated human ventricular cardiomyocyte to assess the effects of mild to extreme hypernatremia on the electrophysiology of such a cardiomyocyte. Together with the hypernatremia-induced decrease in $I_{\text{Kr}}$ and $I_{\text{Ks}}$, increase in $I_{\text{NaCa}}$, and decrease in $I_{\text{NaK}}$, the hypernatremia-induced cell shrinkage hyperpolarizes the resting membrane potential, prolongs the AP, increases the maximum upstroke velocity, and increases the threshold stimulus current at all levels of hypernatremia. These effects are observed over a wide range of beating rates, and their magnitude increases with increasing levels of hypernatremia. In the case of mild to severe hypernatremia, these effects on the electrical activity of human ventricular cardiomyocytes are relatively small. However, the excitability of the ventricular cardiomyocytes is reduced, particularly in the case of extreme hypernatremia.

## Data Availability

The datasets created and analyzed during this study are available from the corresponding author on request.
